# Niobium carbide–mediated photothermal therapy for infected wound treatment

**DOI:** 10.3389/fbioe.2022.934981

**Published:** 2022-11-30

**Authors:** Junyu Ren, Junlong Da, Wei Wu, Ce Zheng, Narisu Hu

**Affiliations:** ^1^ Oral Implant Center, Second Affiliated Hospital of Harbin Medical University, Harbin, China; ^2^ Institute of Hard Tissue Development and Regeneration, Second Affiliated Hospital of Harbin Medical University, Harbin, China; ^3^ Hospital Management Office of Harbin Medical University, Harbin, China

**Keywords:** infected skin, photothermal therapy, nanoparticle, niobium carbide, antibiotics

## Abstract

Bacterial infections of the wounds on the skin surface significantly reduce the rate of wound healing, potentially leading to serious systemic infections. Antibiotics are the first-line drugs for the treatment of these infections. However, the misuse and overuse of antibiotics have led to the emergence of bacterial resistance. Therefore, a new antimicrobial strategy is urgently needed. Photothermal therapy (PTT) is a novel efficient therapeutic technique that can produce irreversible cell damage to induce death of bacteria, possessing a great potential in infected wound healing. This work describes the use of a new photothermal agent (PTA) such as niobium carbide (NbC) nanoparticles with outstanding near-infrared (NIR) absorption property. NbC nanoparticles converted NIR laser irradiation energy into localized heat for photothermal treatment. *In vitro* antimicrobial experiments have revealed that NbC nanoparticles exert excellent antimicrobial effects against *Staphylococcus aureus* (*S. aureus*) and *Escherichia coli* (*E. coli*). Moreover, NbC nanoparticles accelerated *E. coli*–infected wound healing process, reduced inflammatory response, and showed good biosafety *in vivo*. Altogether, NbC nanoparticles represent an efficient PTA for antimicrobial treatment and are a bio-safe material with low toxicity *in vivo*.

## Introduction

The skin is the largest organ in the human body representing the first line of defense and protects the human body from external infestation (Hu et al., 2018; Sheikholeslam et al., 2018). When the skin is damaged by physical or chemical factors, bacteria invade the body and cause infectious diseases (Sorg et al., 2017). Eventually, bacteria can enter the blood, causing severe systemic infections such as pneumonia and septicemia (Sun et al., 2020). Previous studies have suggested that the number of patients in need of treatments for skin and soft tissue infections such as folliculitis and skin abscess has increased since 2000 (Hersh et al., 2008; Klein et al., 2009; Tracy et al., 2011; Morgan et al., 2019). In particular, bacteria living in the “danger triangle,” an area located on the face comprising both the corners of the mouth to the bridge of the nose, may lead to cavernous sinus thrombosis, which is a life-threatening disorder that causes death in humans (DiNubile, 1988; Plewa et al., 2022). In addition, the healing period of the skin can be longer in cases where the patients have other chronic diseases such as diabetes (Wei et al., 2021).

Since bacteria are still the leading cause of skin infections, antibiotics are heavily used to treat these diseases (Nathwani et al., 2016). However, the overuse of antibiotics leads to the development of bacterial resistance (Ko et al., 2018). It is estimated that drug-resistant bacterial infections could kill 10 million people every year by 2050 because of the development of multidrug resistant pathogens (Rai et al., 2017). Therefore, new antibiotics have to be explored to overcome these problems. Unfortunately, the development of antibiotics seriously lags behind the rate of bacterial mutations, which results in antibiotic resistance as a global problem and the need for new antibiotics (Piddock, 2012; Cao et al., 2020). Therefore, a safe, effective, and cost-effective antimicrobial treatment is urgently needed.

Some antibiotic-free techniques, such as photothermal therapy (PTT) and photodynamic therapy, have been developed in recent decades (Son et al., 2020). PTT is a noninvasive treatment with many advantages such as slight bacterial resistance, excellent remote controllability, and low side effects (Mu et al., 2021; Tang et al., 2021). Photothermal agents (PTAs) and near-infrared (NIR) light are crucial in PTT treatment. NIR laser illumination in the range of 700–950 nm improves PTT outcomes. The absorption of laser light in this range by hemoglobin and tissue water decreases, and this improves deeper penetration of the laser light into the tissues (Theune et al., 2019). Moreover, PTAs absorb NIR light and generate localized heat energy, inducing high temperatures around the bacteria and irreversible protein damage, eventually leading to their death (Guo et al., 2013; Nguyen et al., 2015; Xu et al., 2017). Therefore, the photothermal antimicrobial treatment does not induce bacterial resistance, and it is considered to be a safe and an effective antimicrobial modality (Yuwen et al., 2016; Zhang et al., 2019; Huo et al., 2021).

**SCHEME 1:**
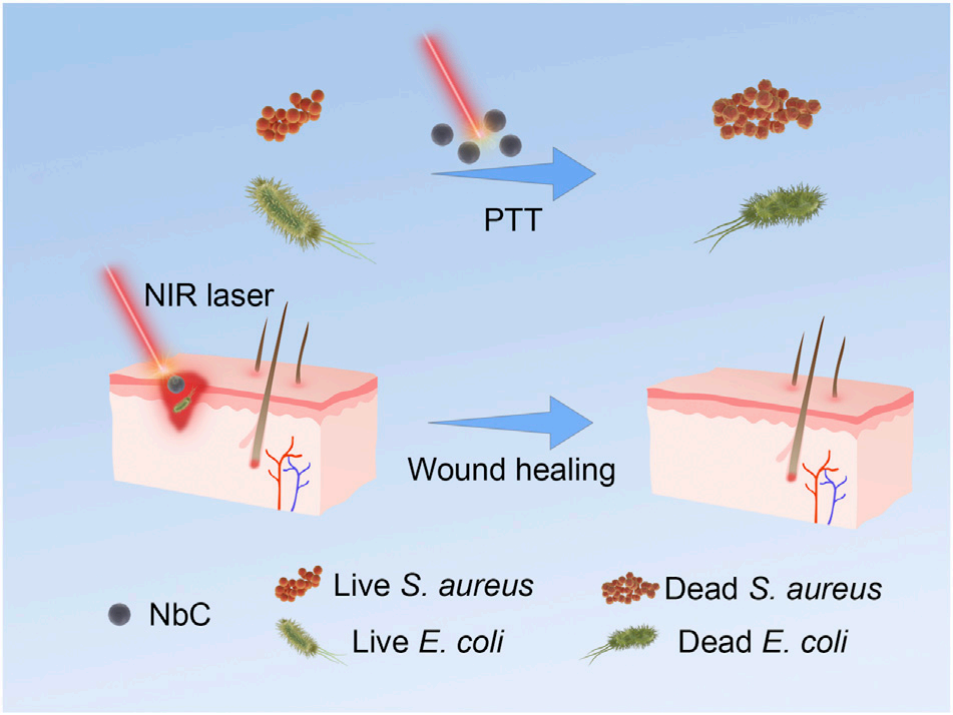
Schematic of NbC nanoparticles for photothermal antimicrobial *in vitro* and the infected-skin treatment.

More and more nanoparticles are used in PTT with the development of nanomaterials science, such as metal nanoparticles, graphene, and organic polymer nanomaterials (Fan et al., 2019; Korupalli et al., 2020; Gupta and Malviya, 2021). Gold nanoparticles are used to kill bacteria by absorbing NIR and producing photothermal effect (Yang et al., 2017). However, their high cost limits their further clinical application. NbC nanoparticles are composed of niobium (Nb) and carbide (C) elements and are expected to have an excellent photothermal effect. However, the research of NbC nanoparticles as a photothermal antimicrobial material has not been reported. Thus, NbC nanoparticles have been selected in this work as a novel photothermal material for *in vivo* photothermal antimicrobial treatment on the infected skin of mice. A variety of bacteria are present on the surface of the skin, including gram-positive (*S. aureus*) and gram-negative (*E. coli*) bacteria (Leong et al., 2018). However, the clinical use of antibiotics is limited by all kinds of conditions and factors. Narrow-spectrum antibiotics do not work on certain types of bacteria. By contrast, broad-spectrum antibiotics have a broader antimicrobial effect but also induce antibiotic resistance (Rao, 1998; Tarrant et al., 2021). Multiple antibiotics are often required for infections caused by a combination of gram-positive and gram-negative bacteria. Therefore, in this work, both gram-positive (*S. aureus*) and gram-negative (*E. coli*) bacteria were used to evaluate the antimicrobial property of NbC nanoparticles to obtain an excellent photothermal material with broad-spectrum antimicrobial properties and negligible antibiotic resistance. Firstly, the characterization of NbC nanoparticles was performed using transmission electron microscopy (TEM), dynamic light scattering (DLS), X-ray diffraction (XRD), and X-ray photoelectron spectroscopy (XPS) to determine whether the size and physical phase meet the requirements of PTAs. An excellent photothermal property was determined that made a significantly contribution to PTT treatment, and therefore the photothermal properties of the NbC nanoparticles were evaluated. An excellent antibacterial effect against gram-positive and gram-negative bacteria is expected for NbC nanoparticles in this work, thus an assay on *S. aureus* and *E. coli* antibacterial effect was performed. Next, the infected skin of mice was used to evaluate the antimicrobial properties of NbC nanoparticles *in vivo*. Finally, the biosafety of these nanoparticles, an important aspect of photothermal materials, was studied by using hematoxylin and eosin (H&E) staining and the Cell Counting Kit-8 (CCK-8) assay to explore biotoxicity *in vivo* and *in vitro* (Scheme 1).

## Material and methods

### Materials

Acridine orange (AO) and ethidium bromide (EB) were purchased from Shanghai Sangon Biotech Company, *o*-nitrophenyl-*β*-*D*-galactoside was purchased from Sigma-Aldrich and the Cell Counting Kit-8 (CCK-8) was purchased from BioMake. Phosphate-buffered solution (PBS), formalin, and deionized water were purchased from Biosharp. Ethanol, Triton X-100, calcein acetoxymethyl ester, propidium iodide, glutaraldehyde, and Luria–Bertani (LB) broth were purchased from Aladdin Company. NbC nanoparticles were synthesized by Shanghai CW NaNo company.

### Characterization

The morphological images of NbC nanoparticles were obtained using TEM (HT7800, Hitachi, Tokyo, Japan). The morphological images of *S. aureus* and *E. coli* were obtained using scanning electron microscope (SEM, SU8010, Hitachi, Tokyo, Japan). The solution absorbance of NbC nanoparticles was recorded on a spectrometer (UV-2600, Shimadzu, Tokyo, Japan). The crystal phase, chemical valence, and composition of NbC nanoparticles were obtained using XRD (X’PERT) and XPS spectra (250Xi, Thermo Scientific, MA, United States). The thermal images of the NbC nanoparticles were taken by a near-infrared camera (FLIR i7, FLIR Systems, Inc., OR, United States) as follows: the infrared camera was aimed at the target, and the shutter was pressed after autofocusing to obtain a clear infrared image. The photothermal effect of NbC nanoparticles was irradiated by NIR laser (808 nm laser, New Industries Optoelectronics Tech, Changchun, China). The live/dead state of *E. coli* and MC3T3-E1 cells was observed on an inverted phase microscope (ECLIPSE Ti, Nikon, Tokyo, Japan).

### Photothermal conversion test

NbC nanoparticle suspensions (750, 500, 250, 125, and 62.5 μg ml^−1^) were prepared to test the photothermal effect of NbC nanoparticles. These solutions containing different concentrations of NbC nanoparticles were added into a quartz cell and irradiated by a NIR-808 with a power of 1 W cm^−2^ for 10 min. The distance between the laser and sample was approximately 1 cm. Finally, the NIR laser was turned off after 10 min of irradiation, and the temperature was recorded over 10 min. The photostability of the NbC nanoparticles was evaluated through a cycle of heating up and cooling down that was repeated thrice using an infrared camera to record the temperature changes every 30 s.

### Cytotoxicity

The *in vitro* toxicity of the NbC nanoparticles was evaluated by hemolysis, CCK-8, and live/dead staining. As regards the hemolysis, the nanoparticles were incubated with 4% (v/v) red blood cells at different concentrations, and the effects on the red blood cells were detected after 2 h. Triton X-100 and PBS were used as positive and negative controls, respectively. As regards the CCK-8 assay, MC3T3-E1 cells were seeded in 96-well plates and incubated at 37°C for 24 h. Next, 200 μl of NbC nanoparticles at different concentrations in the Dulbecco’s modified Eagle’s medium (DMEM) was added to the above wells, which was incubated for another 24 h. Then, MC3T3-E1 cells were treated with 10 μl of CCK-8 and 90 μl of DMEM and incubated for another 4 h. Finally, the absorbance of the different mixtures was read at 450 nm using a microplate reader (Synergy™ HT, BioTek Instruments Inc., United States). As regards the live/dead staining, the cells were washed with PBS buffer and stained with calcein acetoxymethyl ester (calcein AM) and propidium iodide (PI) after culturing them with NbC nanoparticles. The staining results were observed under the fluorescence microscope.

### *In vitro* antimicrobial effect

*S. aureus* and *E. coli* were separately cultured in LB medium overnight (37°C) until they reached the log-phase. Then, *S. aureus* and *E. coli* were washed with PBS buffer three times, and they were diluted in PBS buffer for the subsequent experiments. Equal concentrations of bacteria with 10^4^–10^5^ colony forming units (CFU) ml^−1^ were divided into four groups: 1) untreated control *E. coli* or *S. aureus*, 2) bacteria + NIR-808 group: *E. coli* or *S. aureus* irradiated with NIR-808, 3) bacteria + NbC nanoparticle group: *E. coli* or *S. aureus* treated with NbC nanoparticles, and 4) PTT-treated group: *E.coli* or *S. aureus* treated with NbC nanoparticles + NIR-808. The NbC nanoparticle concentration of the bacteria + NbC nanoparticles group was equal to its concentration in the PTT-treated group (500 μg ml^−1^). The bacteria + NIR-808 group and the PTT-treated group were exposed to NIR-808 laser for 10 min, while the other groups were cultured in the dark. The above groups of bacteria were subjected to a standard plate counting assay to evaluate the antimicrobial ability of NbC nanoparticles. Furthermore, SEM was used to observe the microstructure of *E. coli* and *S. aureus*.

Equal concentrations of bacterial dilutions of the above four groups were slowly applied on agar plates to perform a standard plate counting assay and incubated for 24 h at 37°C. Then, a camera was used to take images of the bacterial colonies. Finally, the number of *S. aureus* or *E. coli* colonies was counted to calculate the viability of bacteria. The formula for calculating the viability of each group of bacteria is as follows (Liu et al., 2019):

$$\text{Viability of bacteria }\left(\text{\%}\right)\;=\;\frac{\text{Nt}}{\text{Nc}}\times 100\text{\%}$$ (1)

where Nt is the bacterial colonies of the test group (bacteria + NIR-808 group, bacteria + NbC nanoparticle group, and PTT treated group), and Nc is the bacterial colonies of the control group.

The SEM images of *S. aureus* and *E. coli* were obtained by the following steps. One milliliter of the *S. aureus* or *E. coli* mixture was centrifuged at 4,000 rpm for 5 min. Then, the *S. aureus* or *E. coli* mixture was washed thrice with PBS and fixed with 2.5% glutaraldehyde for 12 h. Finally, the *S. aureus* or *E. coli* cells were submerged in 30%, 50%, 70%, 80%, 90%, and 100% ethanol for dehydration. Finally, the SEM images were taken on a scanning electron microscope.

### Survival rate of *E. coli* irradiated at different times

Bacteria were cultured with NbC nanoparticles (500 μg ml^−1^) in PBS solution for 3 h to investigate the survival rate of *E. coli* irradiated at different times with NIR-808 laser. The above solutions were exposed to NIR-808 laser at different times, such as 2, 4, 6, 8, and 10 min of illumination with laser. Next, 400 μl of the bacteria was centrifuged at 4,000 rpm for 5 min. Then, the *E. coli* cells were treated with EB and AO staining reagent for the live/dead assay. Finally, 50 μl of equal concentrations of stained bacterial solution was used to observe the live/dead state of *E. coli* on a fluorescence microscope.

SEM observation was performed using another 400 μL of the above solution, which was centrifuged at 4,000 rpm for 5 min. Then, the *E. coli* cells were washed thrice with PBS and fixed with 2.5% glutaraldehyde for 12 h. Subsequently, the *E. coli* cells were submerged in 30%, 50%, 70%, 80%, 90%, and 100% ethanol for dehydration. Finally, the *E. coli* cells were dried and sputter coated with gold, and the SEM images were taken on an SEM.

The leak of *β*-*D*-galactosidase was observed to evaluate the cell membrane integrity of *E. coli*. The *E. coli* cells were divided into the following two groups: 1) *E. coli* + NbC nanoparticles + NIR-808 and 2) *E. coli* + NIR-808. Both groups were exposed to NIR-808 laser at 2, 4, 6, 8, and 10 min. Then, 1 ml of *E. coli* suspension at different illumination times was mixed with 1 ml *o*-nitrophenyl-*β*-*D*-galactoside solution (2.5 mmol L^−1^). Finally, the mixture was added into a quartz cell to test the optical absorption at 420 nm.

### *In vivo* antimicrobial efficiency of niobium carbide nanoparticles

The antimicrobial test was performed on the infected wounds in the mice subjected to PTT treatment to evaluate the *in vivo* antimicrobial efficiency of the NbC nanoparticles. The hair on the back of the babl/c mice was removed, and the skin was disinfected with 75% alcohol thrice. Then, the mice were anesthetized, and an oval wound of approximately 0.5 cm × 0.5 cm was created on the back of the mice. Finally, 100 μl of *E. coli* in PBS at a concentration of 10^8^ CFU ml^−1^ was applied to the abovementioned wound. When the applied *E. coli* in PBS mixture was almost dry, it was reapplied, and the treatment was repeated four times.

The babl/c mice were divided into the following four groups for *in vivo* antibacterial experiments: 1) untreated control mice, 2) NIR-808–irradiated mice, 3) NbC nanoparticles–treated mice, and 4) NbC nanoparticles + NIR-808–treated mice. Fifty milliliters of PBS buffer was added dropwise to the infected wounds in the control and bacteria + NIR-808 groups, while 50 µl of PBS buffer with 500 μg ml^−1^ NbC nanoparticles was added to the wounds in the bacteria + NbC nanoparticles + NIR-808 and bacteria + NbC nanoparticles groups. Bacteria + NIR-808 and bacteria + NbC nanoparticles + NIR-808 groups were irradiated with NIR-808 for 10 min. On the contrary, the untreated control mice and NbC nanoparticles–treated mice were kept in a dark room for 10 min. The wound temperatures were recorded with a near-infrared camera every 30 s. Pictures of the infected skin wounds were taken at 0, 3, 6, 9, and 12 days using a camera. Then, the babl/c mice were sacrificed at day 12, and the wound tissue of the mice was cut with sterile instruments. Finally, these skin tissues were immersed and fixed in 4% formalin solution for 12 h and stained with H&E. Inflammatory cell infiltration was observed under a microscope.

### *In vivo* biosafety study

The changes in the weight of the mice were recorded after different treatments for 12 consecutive days. The blood of the mice was collected to analyze the blood routine indicators. The main organs were collected using sterile instruments at day 12, namely, the heart, liver, spleen, and kidneys. Finally, these organs were fixed in 4% formalin solution, stained with H&E, and observed under the microscope for histological analysis.

## Results and discussion

### Characterization of niobium carbide nanoparticles

**FIGURE 1:**
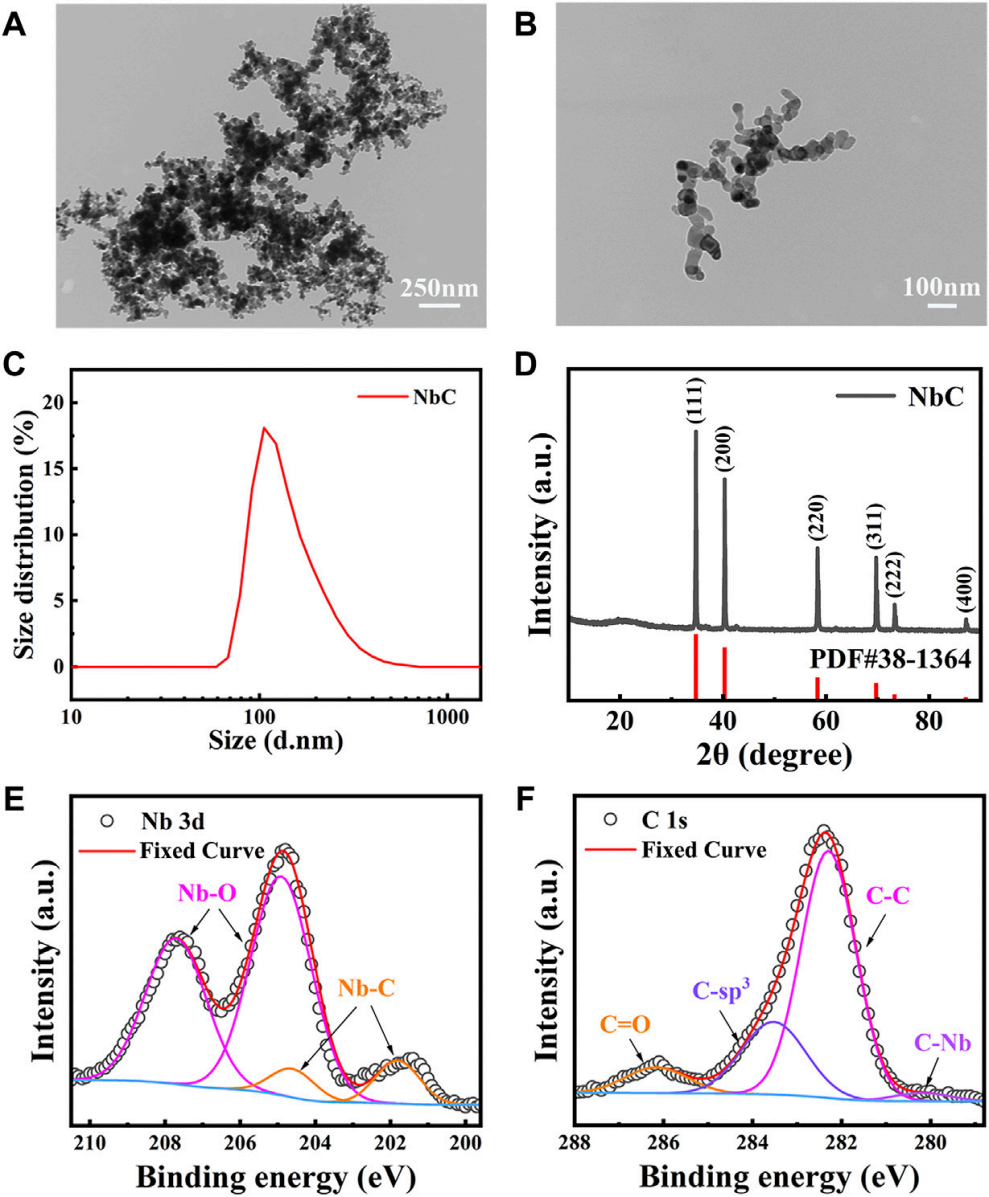
Characterization of NbC nanoparticles. **(A,B)** TEM images of NbC nanoparticles, **(C)** DLS of NbC nanoparticles, **(D)** XRD pattern of NbC nanoparticles, XPS analysis, **(E)** Nb 2p scan of NbC nanoparticles, **(F)** C 1s scan of NbC nanoparticles.

A TEM was used to evaluate the morphological features of the NbC nanoparticles, which are shown in the TEM images in Figures 1A,1B. The average diameter of the NbC nanoparticles was in the range of 20–40 nm, with a maximum dimension size not exceeding 100 nm. The aggregation of the NbC nanoparticles was also observed on the TEM. Then, the DLS results were used to confirm the average NbC particle size (Figure 1C). The hydrated size distribution of the microparticles ranged from 90 to 400 nm. Most of them had an average diameter of 150 nm. This could be due to the fact that the hydrated particle size measured by DLS was slightly larger than the particle size observed with the TEM. The NbC nanoparticles aggregated during the test, as shown in Figure 1A.

The crystal nature of the NbC nanoparticles was investigated by XRD. Figure 1D shows all the XRD characteristic Bragg peaks of the NbC nanoparticles, which are consistent with the standard card PDF #38-1364 for NbC nanoparticles, and no impurities were found. The crystallite size was 52.6 nm, as calculated according to the Debye–Scherrer equation from the XRD spectra. No evident change in the structure of the NbC nanoparticles was observed after laser irradiation (Supplementary Figure S1). Figures 1E,F show the X-ray photoelectron spectroscopy full spectrum data revealing the narrow scan analysis of the Nb and C elements in the NbC nanoparticles. The spectra of Nb 3d (Figure 1E) shows Nb 3d orbitals split g into two parts, namely, 3d 3/2 and 3d 5/2, due to the interaction between the spin orbitals, and the energy difference was approximately 2.8 eV. A quadruple peak was fitted in the range of 199.6–210.5 eV, where the XPS signal peaks at 207.71 and 204.91 eV were attributed to the characteristic XPS signal peaks of +2 valence Nb 3d 3/2 and 3d 5/2 in the Nb-C bond, respectively; the two peaks at 204.61 and 201.81 eV corresponded to the XPS signal peaks of the higher valence Nb-O bond of the XPS signal peaks. The adsorption of oxygen on the surface of NbC nanoparticles led to the presence of the Nb-O peak. XPS spectra of C 1s in Figure 1F showed the presence of a C-C bond and free carbon at 282.29 and 283.53 eV. The presence of C-Nb at 280.18 eV was associated with the formation of NbC nanoparticles. Besides, the presence of a C=O peak at 286.16 eV indicated the adsorption of oxygen on the sample surface. The above XRD and XPS results confirmed the purity of the NbC nanoparticles sample. The sample inevitably contacted with air, and the C=O peak was detected during the test. In conclusion, the NbC nanoparticles were of high purity without other impurities, with an average diameter of 150 nm, which met the physical phase requirements of PTAs.

### Optical behavior and photothermal properties of niobium carbide nanoparticles

**FIGURE 2:**
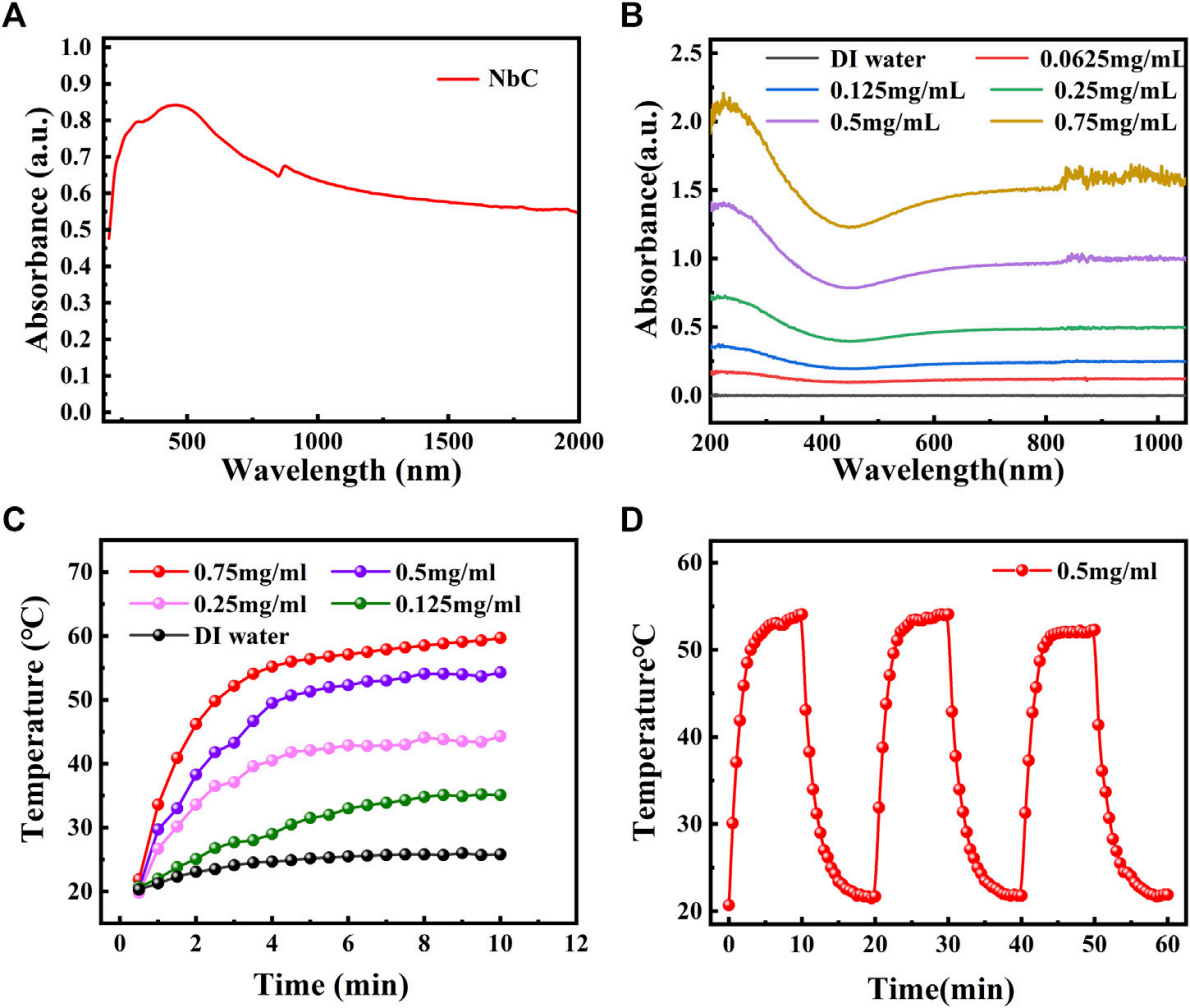
The optical absorbance and photothermal properties of NbC nanoparticles. **(A)** The optical absorbance of solid powder of NbC nanoparticles and **(B)** the optical absorbance of NbC nanoparticles aqueous solution. **(C)** Heating curve of NbC nanoparticles aqueous solution with 1 W cm^−2^ NIR-808 irradiation (62.5, 125, 250, 500, and 750 μg ml^−1^). **(D)** Heating up and cooling down assay for NbC nanoparticles aqueous solution (500 μg ml^−1^, 1 W cm^−2^).

The property of NIR laser absorption is a prerequisite for using NbC nanoparticles as a PTA for photothermal antimicrobial treatment. The powder absorbance of the NbC nanoparticles was first studied. The NbC nanoparticles had an excellent optical absorption, ranging from 200 to 2,000 nm in the ultraviolet–visible–near-infrared (UV-Vis-NIR) field, which covered the first NIR biological window (the region of 700–980 nm) and the second NIR biological window (the region of 1,000–1,400 nm) as shown in Figure 2A. Secondly, the solid powder of the NbC nanoparticles was dispersed into an aqueous solution to make a solution at the concentrations of 62.5, 125, 250, 500, and 750 μg ml^−1^. The NbC nanoparticles' dispersion at different concentrations showed a strong absorption from 200 to 1,000 nm, and the absorbance was concentration dependent (Figure 2B). It is worth noting that the aqueous solution of NbC nanoparticles at a concentration of 500 μg ml^−1^ had high absorbance in the range of 700–1,000 nm. Thus, this concentration in aqueous solution was selected for subsequent experiments.

NbC nanoparticle dispersion was exposed to the NIR-808 laser (1 W cm^−2^) for 10 min to evaluate the photothermal properties of the NbC nanoparticles. The dispersion at different concentrations showed an outstanding photothermal effect under NIR-808 irradiation, in which the highest temperature was 53.8 °C when the concentration of 500 μg ml^−1^ was used (Figure 2C). Moreover, the NbC nanoparticles dispersion showed a fantastic photothermal stability after subjecting them to three cycles of heating up and cooling down (Figure 2D). These results confirmed that the NbC nanoparticles had outstanding photothermal properties for their potential use for photothermal antimicrobial treatment.

### *In vitro* photothermal therapy antimicrobial property

**FIGURE 3:**
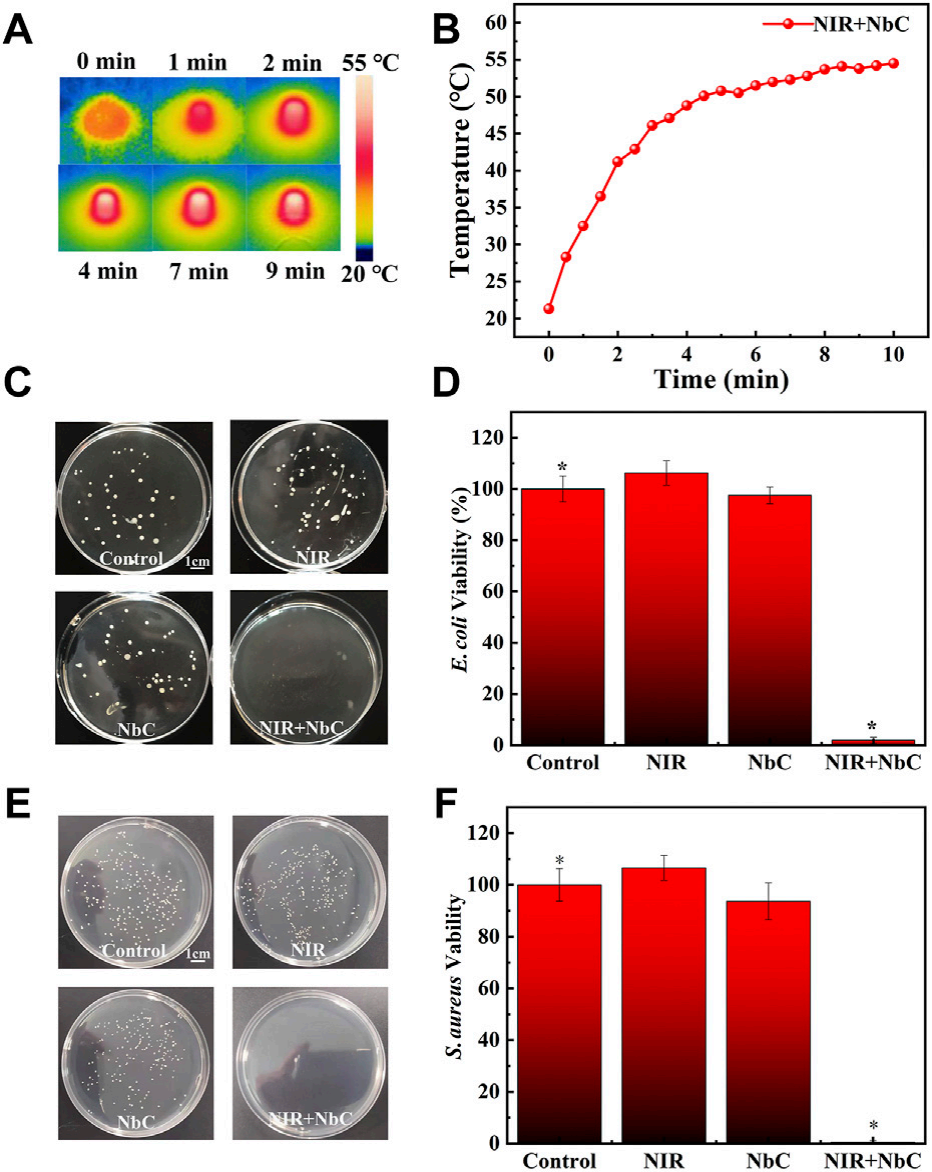
The effect of NbC nanoparticles with laser irradiation on killing of bacteria. **(A,B)** Thermographic images and heating curve of NbC nanoparticles bacterial suspension with a concentration of 500 μg ml^−1^. **(C,D)** The standard plate counting images and survival rate curve of *E. coli* treated with different methods. **(E,F)** The standard plate counting images and survival rate curve of *S. aureus* treated with different methods (n = 4, *p < 0.05).

The photothermal antimicrobial ability of NbC nanoparticles was evaluated by performing antimicrobial experiments on *E. coli* and *S. aureus*. The thermographic images of NbC nanoparticles in bacterial solution were recorded every 30 s (Figure 3A). The bacterial suspension containing NbC nanoparticles (500 μg ml^−1^) showed a rapid increase in temperature under NIR laser irradiation (Figure 3B). The PTT antimicrobial ability of the NbC nanoparticles was evaluated using an equal concentration of *E. coli* or *S. aureus* in PBS suspension, using the following groups: 1) untreated control *E. coli* or *S. aureus*, 2) *E. coli* or *S. aureus* irradiated with NIR-808 laser, 3) *E. coli* or *S. aureus* treated with NbC nanoparticles, and 4) *E. coli* or *S. aureus* treated with NbC nanoparticles + NIR-808 (PTT treated group). Figure 3C shows the standard plate counting images of different *E. coli* groups. Then, the number of colonies of *E. coli* was counted and the survival rates of *E. coli* were calculated. The survival rate of *E. coli* in the NbC nanoparticles and NIR-treated groups was not significantly decreased when compared with that of the control group. By contrast, the number of colonies and survival rate in the PTT-treated group were significantly reduced when compared with those in the other three groups (Figure 3D). No significant reduction in bacterial colony numbers and survival rate was observed in the NIR-treated group or NbC nanoparticles–treated group, indicating that the use of NIR-808 laser or NbC nanoparticles alone did not affect the growth of *E. coli*. Similar experimental results were observed using *S. aureus* (Figures 3E,F). The survival rate of *S. aureus* was reduced in the PTT-treated group when compared to that of the NIR-808 irradiation group, NbC nanoparticle treatment group, and untreated control group (*n* = 4, *p* < 0.05). According to the aforementioned results, the temperature of the NbC nanoparticles suspension irradiated with NIR-808 laser exceeded 50°C, which is warmer than the temperature necessary for protein denaturation. The bacterial membrane is composed of many proteins performing various functions on the cell membrane (Lepock et al., 1987; Bos et al., 2004; Yang et al., 2019). Thus, the bacterial suspension treated with NbC nanoparticles + NIR-808 generated a localized high temperature around *E. coli* and *S. aureus*, leading to protein denaturation in the bacterial cell membrane.

**FIGURE 4:**
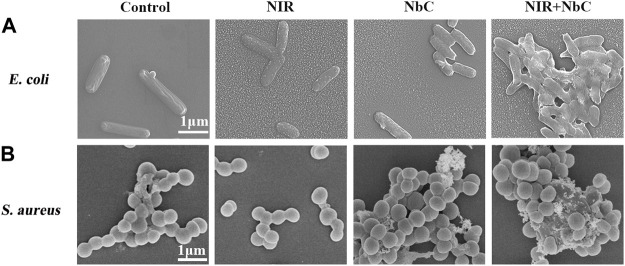
The SEM images of *E. coli* and *S. aureus* treated with different methods. SEM images of **(A)** *E. coli* and **(B)** *S. aureus*—untreated control group, irradiated with NIR-808 group, NbC nanoparticles–treated group, and PTT-treated group.

SEM observations were performed to further examine the photothermal antimicrobial property of the NbC nanoparticles. *E. coli* in the untreated control group showed a uniform cylindrical shape with a length of 2–3 μm and a width of 0.5–1 μm and had a fully shaped smooth cell membrane without cytoplasmic outflow, with all characteristics similar to those of *E. coli* in the NIR-808–irradiated and NbC nanoparticles–treated groups (Figure 4A). However, after 10 min of treatment with NbC nanoparticles + NIR-808, the morphology of *E. coli* became flattened, and the bacterial cell membrane was disrupted and blurred. Similarly, *S. aureus* cells in the control group had a spherical shape, as revealed by the SEM, with a diameter ranging from 0.5 to l μm and a smooth and intact cell membrane (Figure 4B). Nevertheless, the shape of *S. aureus* significantly changed after 10 min of PTT treatment, the cells had shrunk, and the membrane showed apparent folds. However, a proportion of *S. aureus* was still in good condition after PTT treatment. Consequently, the SEM observation revealed that PTT directly triggered *E. coli* and *S. aureus* death by cell membrane damage. Moreover, NbC nanoparticle–mediated PTT had an excellent and greater damage ability on the membrane of *E. coli* than of *S. aureus*. This result might be explained by the difference in structure of the cell membranes of *E. coli* and *S. aureus.* Indeed, the cell membrane of gram-negative bacteria consists of a lipid bilayer, which is composed of a large amount of outer membrane proteins. By contrast, the cell wall of gram-positive bacteria contains little amounts of protein and is mainly composed of peptidoglycan and teichoic acid (van Loosdrecht et al., 1987; Scheffers and Pinho, 2005; Yang et al., 2019). The outer membrane contains proteins sensible to heat, thus the phototherapeutic effect of the NbC nanoparticles was more effective on the cell membrane of *E. coli* than of *S. aureus* (Burgess et al., 2008; Yang et al., 2019).

### Survival rate of *E. coli* irradiated at different times

**FIGURE 5:**
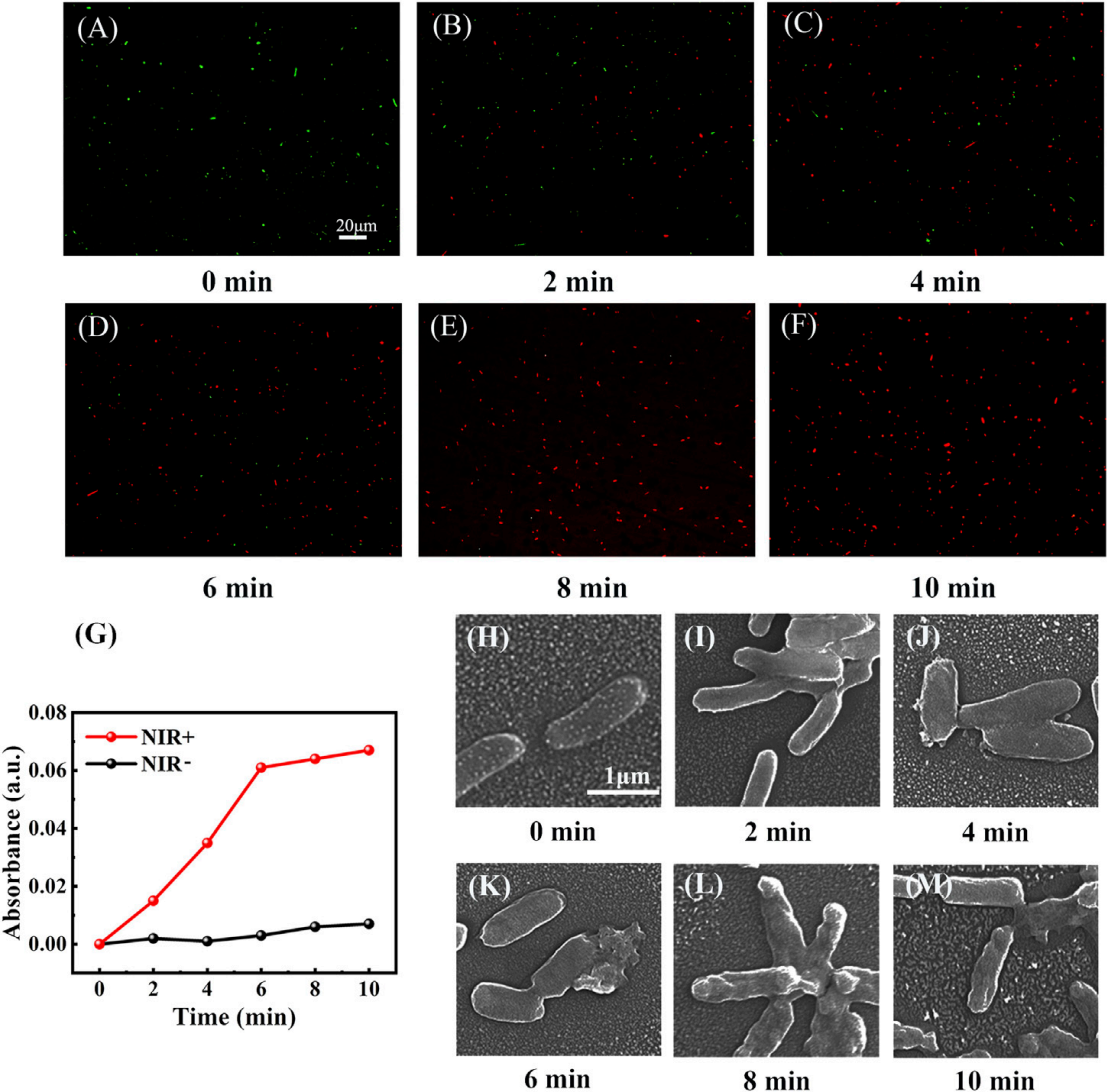
*E. coli* irradiated with NbC nanoparticles at different times. **(A–F)** Live/dead staining of *E. coli* irradiated with NIR-808 laser after 2, 4, 6, 8, and 10 min. **(G)** The production of *o*-nitrophenol versus irradiation time. **(H–M)** SEM images of *E. coli* irradiated at different times (2, 4, 6, 8, and 10 min).

A gradual increase in multiple antibiotic resistance poses a challenge to the treatment of infected skin wounds (Zhu et al., 2021). In recent years, gram-negative multidrug-resistant bacteria have attracted disproportionate attention, but despite that, currently no commercially available antibiotics are available to combat them (Levy and Marshall, 2004; Zgurskaya et al., 2015; Zhu et al., 2021). Therefore, *E. coli* PBS suspension containing equal concentrations of NbC nanoparticles (500 μg ml^−1^) was irradiated with NIR-808 (1 W cm^−2^) for 2, 4, 6, 8, and 10 min to further investigate the antimicrobial effect of NbC nanoparticles on gram-negative bacteria after irradiation at different times. The antimicrobial activity of different NIR irradiation time was evaluated by the live/dead staining assay. Dead *E. coli* cells were stained red with the EB staining agent, while live *E. coli* cells were stained green with AO in the live/dead staining. When the irradiation time of NIR-808 was 0 min, the vast majority of the spots were stained green and a few were stained red, as shown in Figure 5A. After 2 min, some *E. coli* was stained red, indicating that they had died (Figure 5B). It is worth noting that the number of red spots increased with increasing irradiation time (Figures 5C,D). When the time was enhanced to 8 min, most *E. coli* were stained red when observed under a fluorescence microscope (Figure 5E). Almost all *E. coli* appeared to be stained red at 10 min, as shown in Figure 5F, suggesting that almost all *E. coli* were dead. These results have indicated that the viability rate of *E. coli* decreased with increasing irradiation time. This result has been confirmed by performing the galactosidase activity assay. *β*-*D*-galactosidase is one of the cytoplasmic components of *E. coli* that catalyzes the production of light green *o*-nitrophenol from *o*-nitrophenol-*β*-*D*-galactoside. *β*-*D*-galactosidase is stored inside *E. coli* cells, and *β*-*D*-galactosidase can flow outside the cell only when the cell membrane is damaged, promoting *o*-nitrophenol production, which indicates death of *E. coli* cells. Figure 5G shows that the curve of *o*-nitrophenol *versus* the irradiation time confirms that NbC nanoparticles–mediated PTT treatment continuously reduced the viability rate of *E. coli* with the enhancement of the irradiation time.

The abovementioned results have been further confirmed by observing *E. coli* at different treatment times by scanning electron microscopy. The appearance of unirradiated *E. coli* was smooth, with an intact cell membrane (Figure 5H). Cell membrane disruption in *E. coli* was observed after 2 min of NIR irradiation. However, the shape of *E. coli* did not significantly change, and the number of *E. coli* cells with a disrupted membrane was not abundant at 2 min (Figure 5I). Next, *E. coli* with disrupted cell membranes was observed under scanning electron microscopy in all groups treated with light irradiation at different times (Figures 5J–M). Interestingly, an outflow of cytoplasm from *E. coli* was observed at 6 min (Figure 5K). These SEM images further confirmed that the cell membrane of *E. coli* was indeed disrupted, and the cell content flowed out, finally inducing death of *E. coli* cells after different NIR light exposure times. In summary, the mortality of *E. coli* was directly proportional to the radiation time. Although the damage to the membranes of *E. coli* cells was observed under SEM in these four groups (Figures 5I–M), what is regrettable was that we did not observe whether the severity of disruption of the cell membrane structures was influenced by different NIR-808 irradiation times, as observed for the cell membrane integrity and cell volume.

### Photothermal therapy antimicrobial effect in babl/c mice

**FIGURE 6:**
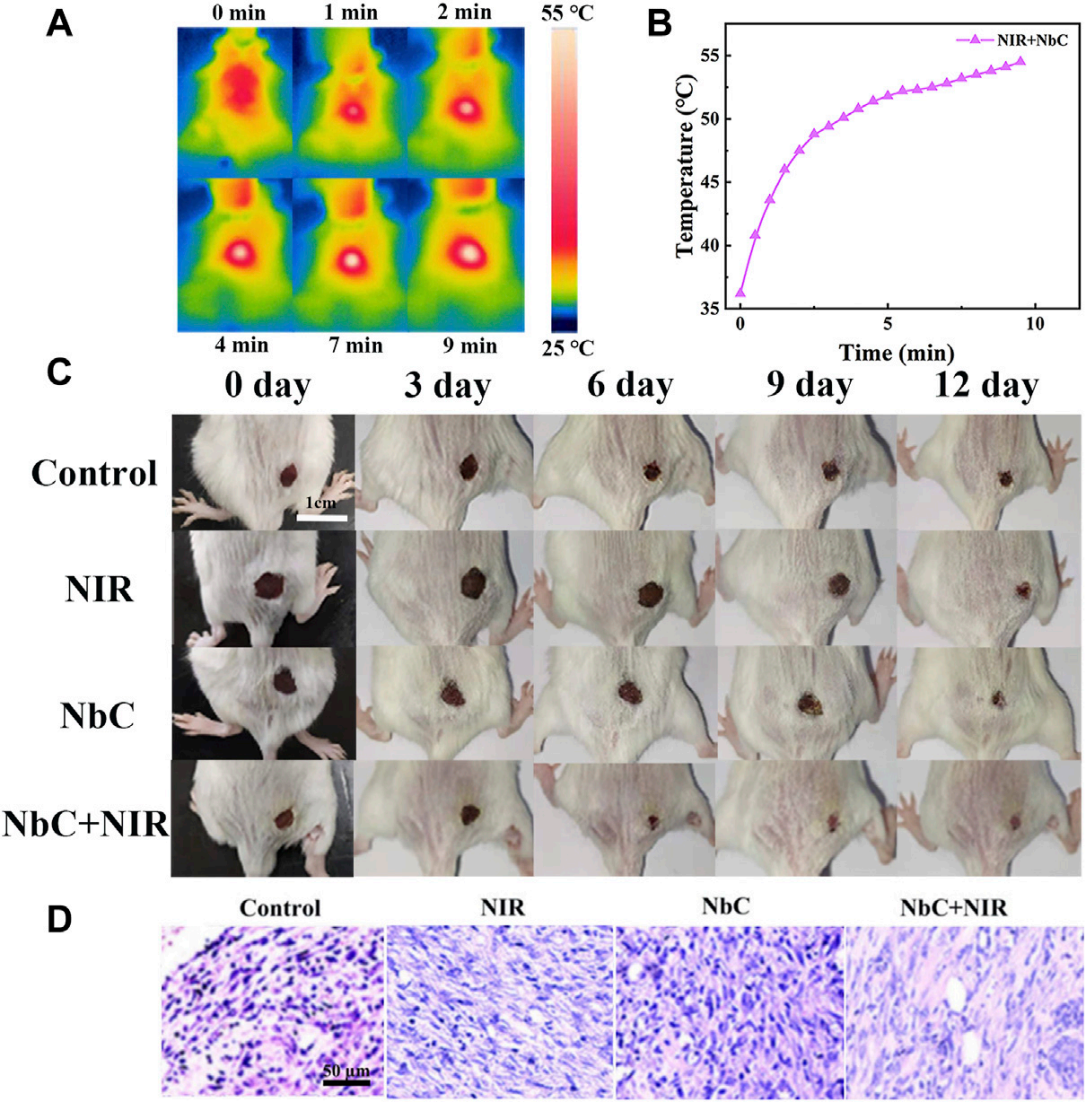
Photothermal antimicrobial treatment of infected skin in babl/c mice. **(A)** Photothermal image of mice after laser irradiation at 0–10 min, and **(B)** photothermal curve of mice with irradiation. **(C)** The healing process of the infected skin wound after different treatments at 0, 3, 6, 9, and 12 days. **(D)** H&E staining image of the skin at 12 days.

The PTT antimicrobial effect of the NbC nanoparticles was also evaluated *in vivo* on babl/c mice. Firstly, an oval wound of approximately 0.5 cm × 0.5 cm was created on the back of the mice. Hundred milliliters of *E. coli* bacterial PBS solution with a concentration of 10^8^ CFU ml^−1^ was applied to the wound. Then, the mice were divided into the following four groups: 1) untreated control mice, 2) NIR-808–irradiated mice, 3) NbC nanoparticles–treated mice, and 4) NbC nanoparticles + NIR-808–treated mice. The temperature of the skin wounds was observed using a near-infrared camera to monitor the temperature during the photothermal antimicrobial process. Figures 6A,6B shows that the temperature of the skin wounds was over 50°C with NbC nanoparticles–mediated NIR-808 irradiation, which is sufficient to cause death in *E. coli* (Yang et al., 2019). After that, wound healing was observed daily and recorded with a camera. A scab was first formed on the surface of the skin wound infected with bacteria after the treatment with NbC nanoparticles + NIR-808 for 3 days (Figure 6C). The wound area of the mice was significantly reduced after 6 days when compared to the untreated control, NbC nanoparticles–treated mice, and NIR-808–irradiated group. Most of the scabs had disappeared from the surface of the skin wounds after 9 days in the NbC nanoparticles + NIR-808–treated mice, and the wounds showed signs of healing. However, scabs were still visible on the wound surface of the mice of the other three groups. The wounds of NbC nanoparticles + NIR-808–treated mice had largely healed after 12 days. The wound areas of the other three groups were also significantly reduced, but still covered with scabs. Thus, the *in vitro* antimicrobial effect is as follows: NbC nanoparticles + NIR-808–treated mice > untreated control group, NIR-808–irradiated group, and NbC nanoparticles–treated group. In summary, NbC nanoparticles–mediated PTT shortened the healing time of the infected skin through the induction of death of *E. coli* on the wound surface.

H&E staining was performed to assess the inflammatory status of the tissue surrounding the infected skin region after healing for 12 days to further evaluate the effect of PTT antimicrobial effect. Many inflammatory cells surrounding the blood vessels were found in the untreated control, NIR-808–irradiated mice, and NbC nanoparticles–treated mice (Figure 6D). However, inflammatory cells were significantly reduced in the NbC nanoparticles + NIR-808–treated mice, suggesting that the infected skin of the mice subjected to PTT had a better healing effect than those in the other three groups. This result further demonstrated that the NbC nanoparticles + NIR-808 effectively promoted the healing of the infected wound. Thus, NbC nanoparticles have great potential in performing an effective photothermal antimicrobial therapy.

### Biosafety of the niobium carbide nanoparticles

**FIGURE 7:**
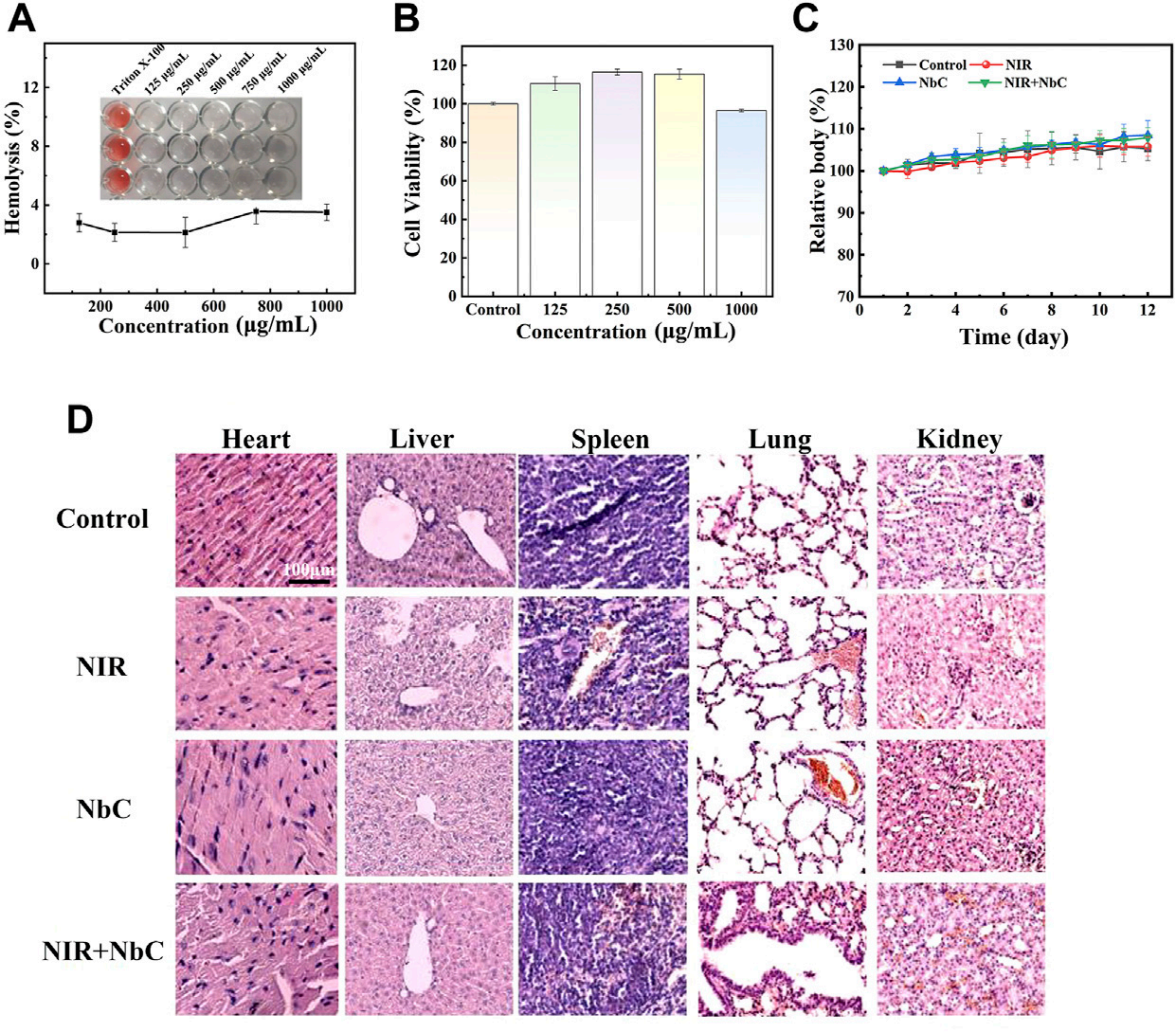
Bio-safety test of the NbC nanoparticles *in vitro* and *vivo*. **(A)** Hemolysis test results of the red blood cells. **(B)** CCK-8 assay *in vitro*. **(C)** Body weight curve of mice during the treatment. **(D)** Histological analysis of major organs of mice.

A good biosafety is a crucial requirement for PTAs. Thus, the *in vitro* and *in vivo* bio-toxicity of the NbC nanoparticles was also assessed. The results of the hemolysis test on red blood cells showed that the hemolysis rates of different concentrations of NbC nanoparticles were below 5% (Figure 7A). The CCK-8 assay was used to examine the *in vivo* biosafety. MC3T3-E1 cells were cultured with different concentrations of NbC nanoparticles (125, 250, 500, and 1,000 μg ml^−1^) in well plates for 4 h. After that, the relative cell viability of the MC3T3-E1 cells was tested. The survival rate of the MC3T3-E1 cells remained above 96% when cultured with different concentrations of NbC nanoparticles suspension from 125 μg ml^−1^–1,000 μg ml^−1^ (Figure 7B). The cell survival rate of the MC3T3-E1 cells was 96.48% also when the MC3T3-E1 cells were cultured with high concentrations of NbC nanoparticles (1,000 μg ml^−1^). The results of live/dead staining confirmed the results of CCK-8. The green cells represent living cells, while the red cells represent dead cells. Almost no dead cells were found within the concentration range of the experiment. This result demonstrated once again that these nanoparticles had excellent cytocompatibility (Supplementary Figure S2). Then, the weight changes of the mice during the treatment were recorded. The body weight of the mice in these four groups increased differently in the different groups, and no significant decrease was observed (Figure 7C). The blood results of the mice showed that a series of blood test indicators were within the normal range after the PTT treatment (Supplementary Figure S3). Finally, the H&E staining on the main organs revealed no significant difference among these four groups (Figure 7D). These results showed that NbC nanoparticles hardly exert cell and tissue toxicity *in vitro* and *in vivo*.

## Conclusion

This work evaluated the photothermal efficiency and photothermal antimicrobial property of the NbC nanoparticles. The SEM and DLS results showed that the average diameter size of the NbC nanoparticles met the basic requirements for PTT. In addition, XRD and XPS confirmed that the NbC nanoparticle samples were of high purity without any impurities, and the NbC nanoparticles powder and suspension had a relatively intense optical absorption in the NIR region according to the optical behavior test. NbC nanoparticles showed an excellent photothermal conversion effect and induced death in *E. coli* and *S. aureus* through the disruption of the cell membranes *in vitro*. However, NbC nanoparticles–mediated PTT treatment exerted a stronger damage on *E. coli* than on *S. aureus*, which might be attributed to the different structures of the cell membrane. The *in vivo* antimicrobial test confirmed that the NbC nanoparticles–mediated photothermal antimicrobial treatment effectively promoted the healing speed of the infected skin wounds in babl/c mice. More importantly, NbC nanoparticles were a good bio-safe material with low toxicity *in vivo* and *in vitro*. However, many difficulties still exist in applying NbC nanoparticles in clinical therapy, since the long-term biological toxicity of NbC nanoparticles have to be evaluated. Our study is of great value for further exploring the application of NbC nanoparticles in clinical antimicrobial therapy in the future. On the one hand, the photothermal conversion efficiency of NbC could be improved by continuous modification as compared with other nanoparticles applied for PTT, and on the other hand, antibacterial drugs other than antibiotics could be loaded to improve the antibacterial effect. The continuous development of biomedicine and the increasing knowledge of micro- and nanomaterials may make it possible for NbC nanoparticles to become an effective alternative for traditional antibiotics, as NbC nanoparticles are an excellent PTT antimicrobial material with low toxicity.

## Data Availability

The original contributions presented in the study are included in the article/Supplementary Material, and further inquiries can be directed to the corresponding author.

## Supplementary material

Supplementary Figure S1 XRD spectra of NbC nanoparticles after laser irradiation.

Supplementary Figure S2 The live/dead staining images of MC3T3 E1 cell after culture with different concentrations of NbC nanoparticles.

Supplementary Figure S3 The main blood routine indexes of mice. White blood cells (WBC), lymphocytes (LYM), monocytes (MON), red blood cells (RBC), hemoglobin (HGB), mean corpuscular hemoglobin (MCH), hematocrit (HCT), mean corpuscular volume (MCV), mean corpuscular hemoglobin concentration (MCHC), red blood cell distribution width (RDW), platelet (PLT), mean platelet volume (MPV), plateletcrit (PCT), platelet distribution width (PDW), neutrophil granulocyte (GRA).
