# Supplementary figures and images for: Niobium carbide–mediated photothermal therapy for infected wound treatment
Source: Front Bioeng Biotechnol. 2022 Nov 30;10:934981. doi: 10.3389/fbioe.2022.934981 (PMC9748739; doi:10.3389/fbioe.2022.934981)

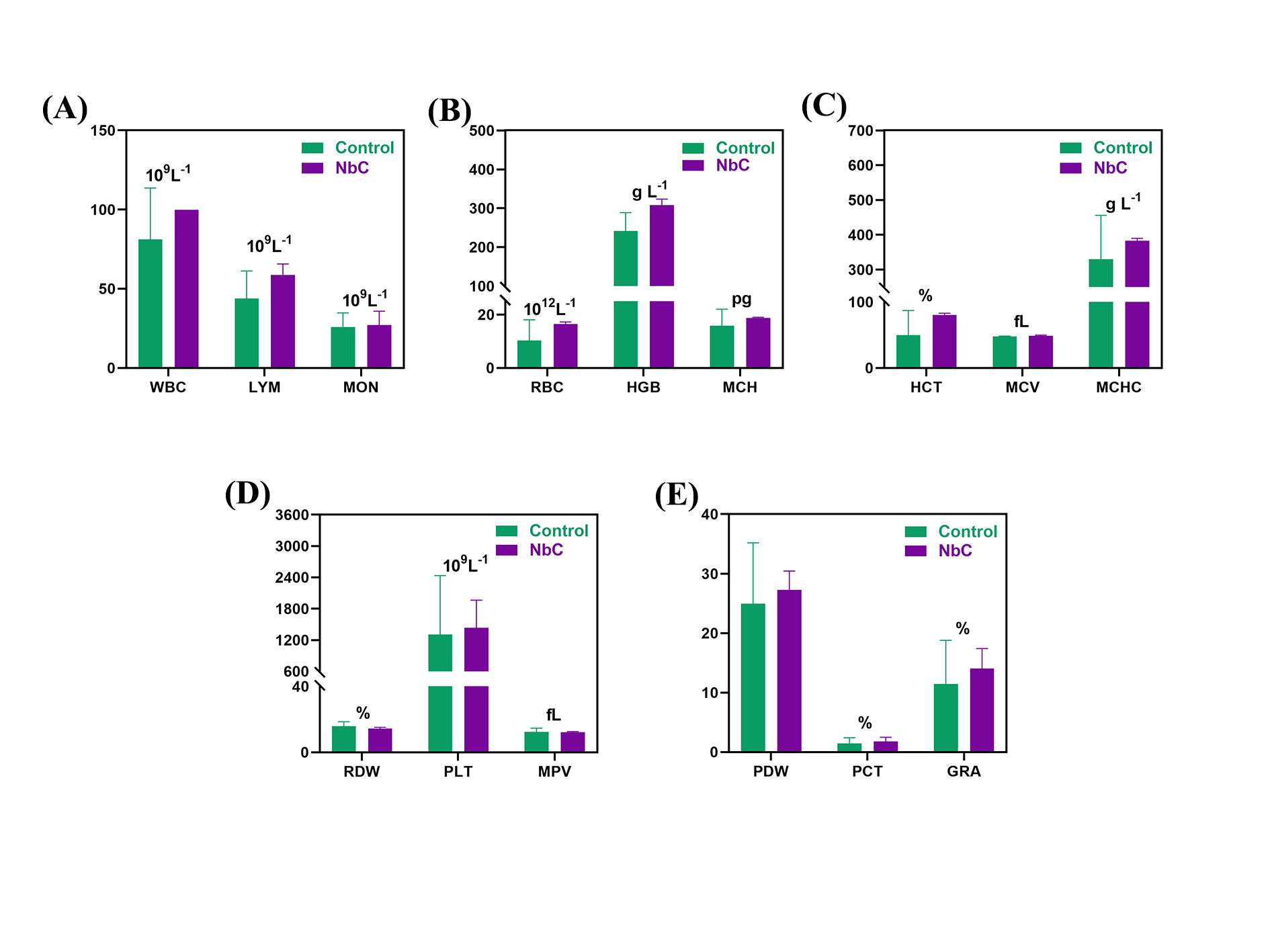

Supplement: Supplementary file 1 [file Image3.TIF]

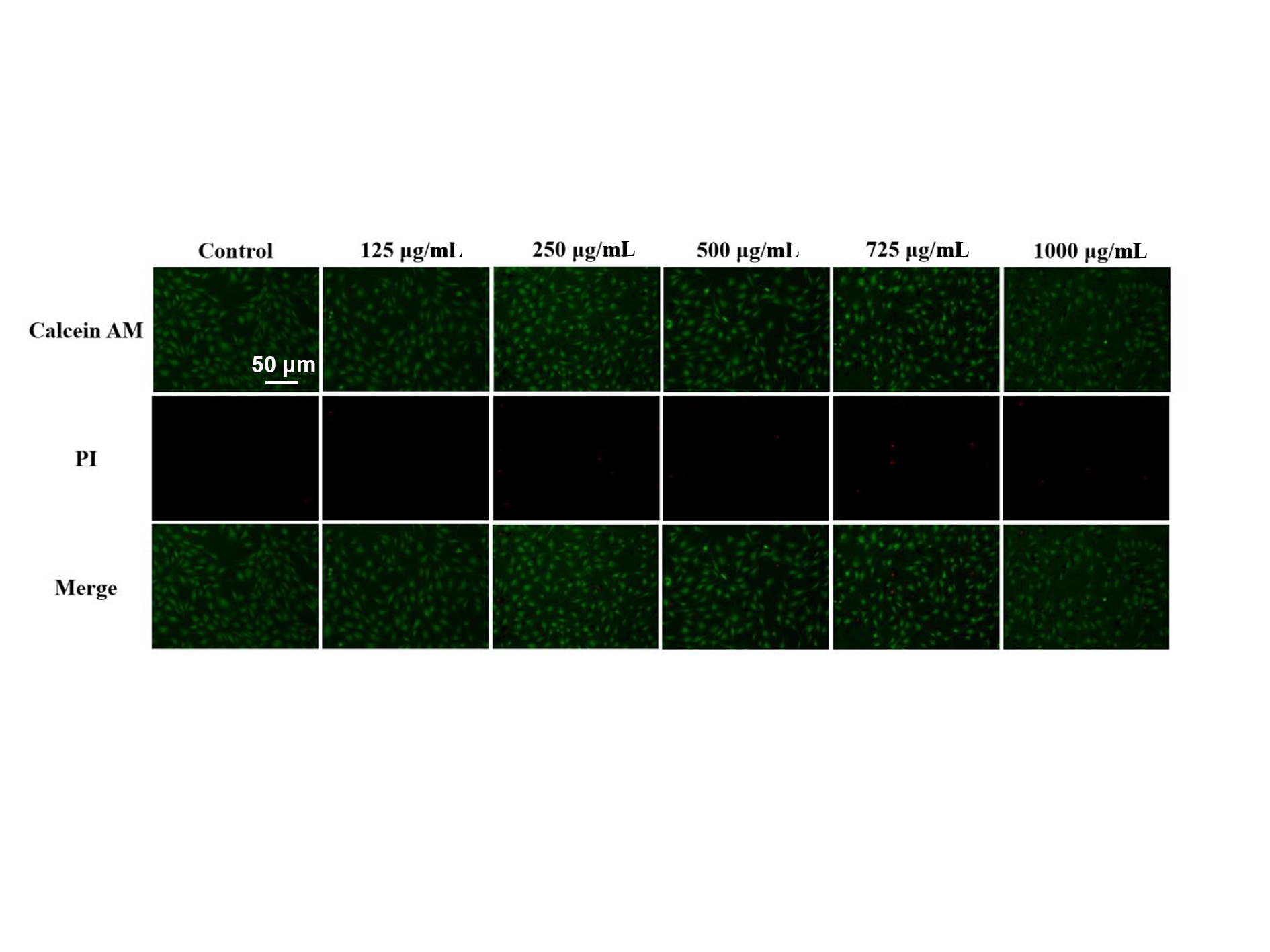

Supplement: Supplementary file 2 [file Image2.TIF]

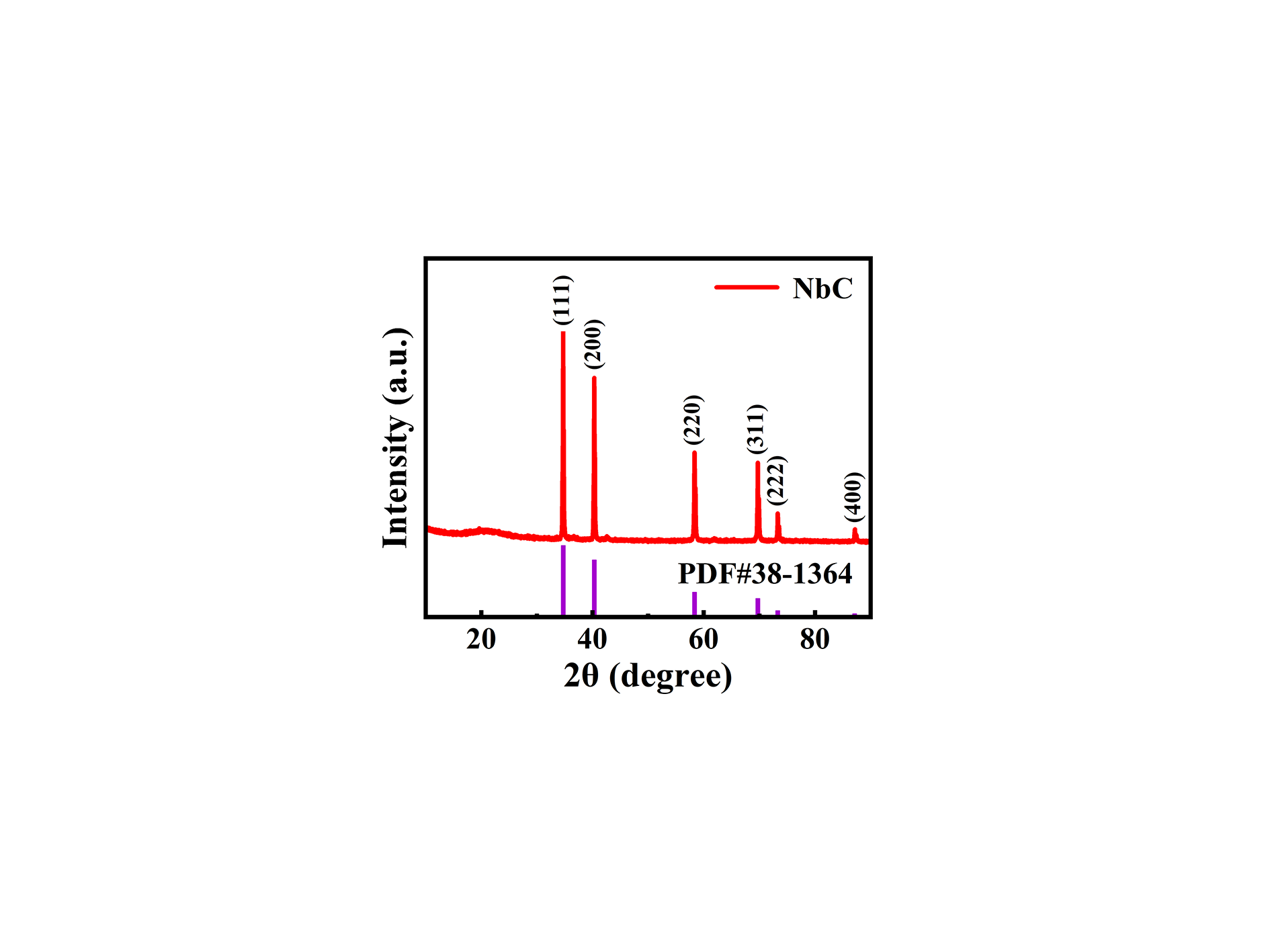

Supplement: Supplementary file 3 [file Image1.TIF]
